# Implementing COVID-19 (SARS-CoV-2) Rapid Diagnostic Tests in Sub-Saharan Africa: A Review

**DOI:** 10.3389/fmed.2020.557797

**Published:** 2020-10-30

**Authors:** Jan Jacobs, Vera Kühne, Octavie Lunguya, Dissou Affolabi, Liselotte Hardy, Olivier Vandenberg

**Affiliations:** ^1^Department of Clinical Sciences, Institute of Tropical Medicine Antwerp, Antwerp, Belgium; ^2^Department of Microbiology, Immunology and Transplantation, KU Leuven, Leuven, Belgium; ^3^Department of Clinical Microbiology, National Institute of Biomedical Research, Kinshasa, Democratic Republic of Congo; ^4^Microbiology Unit, Department of Clinical Biology, University Hospital of Kinshasa, Kinshasa, Democratic Republic of Congo; ^5^Clinical Microbiology, Centre National Hospitalier et Universitaire Hubert Koutoukou MAGA, Cotonou, Benin; ^6^Center for Environmental Health and Occupational Health, School of Public Health, Université Libre de Bruxelles (ULB), Brussels, Belgium; ^7^Innovation and Business Development Unit, Laboratoire Hospitalier Universitaire de Bruxelles - Universitair Laboratorium Brussel (LHUB-ULB), ULB, Brussels, Belgium; ^8^Division of Infection and Immunity, Faculty of Medical Sciences, University College London, London, United Kingdom

**Keywords:** COVID-19, diagnostics, low-resource settings, sub-Saharan Africa, rapid diagnostic tests (RDT), SARS—CoV-2

## Abstract

**Introduction:** For the COVID-19 (SARS-CoV-2) response, COVID-19 antigen (Ag), and antibody (Ab) rapid diagnostic tests (RDTs) are expected to complement central molecular testing particularly in low-resource settings. The present review assesses requirements for implementation of COVID-19 RDTs in sub-Saharan Africa.

**Methods:** Review of PubMed-published articles assessing COVID-19 RDTs complemented with Instructions for Use (IFU) of products.

**Results:** In total 47 articles on two COVID-19 Ag RDTs and 54 COVID-19 Ab RDTs and IFUs of 20 COVID-19 Ab RDTs were retrieved. Only five COVID-19 Ab RDTs (9.3%) were assessed with capillary blood sampling at the point-of-care; none of the studies were conducted in sub-Saharan Africa. *Sampling:* Challenges for COVID-19 Ag RDTs include nasopharyngeal sampling (technique, biosafety) and sample stability; for COVID-19 Ab RDTs equivalence of whole blood vs. plasma/serum needs further validation (assessed for only eight (14.8%) products). *Sensitivity—Specificity*: sensitivity of COVID-19 Ag and Ab RDTs depend on viral load (antigen) and timeframe (antibody), respectively; COVID-19 Ab tests have lower sensitivity compared to laboratory test platforms and the kinetics of IgM and IgG are very similar. Reported specificity was high but has not yet been assessed against tropical pathogens. *Kit configuration:* For COVID-19 Ag RDTs, flocked swabs should be added to the kit; for COVID-19 Ab RDTs, finger prick sampling materials, transfer devices, and controls should be added (currently only supplied in 15, 5, and 1/20 products). *Usability and Robustness: s*ome COVID-19 Ab RDTs showed high proportions of faint lines (>40%) or invalid results (>20%). Shortcomings were reported for buffer vials (spills, air bubbles) and their instructions for use. *Stability:* storage temperature was ≤ 30°C for all but one RDT, in-use and result stability were maximal at 1 h and 30 min, respectively. *Integration in the healthcare setting* requires a target product profile, landscape overview of technologies, certified manufacturing capacity, a sustainable market, and a stringent but timely regulation. In-country deployment depends on integration in the national laboratory network.

**Discussion/Conclusion:** Despite these limitations, successful implementation models in triage, contact tracing, and surveillance have been proposed, in particular for COVID-19 Ab RDTs. Valuable experience is available from implementation of other disease-specific RDTs in sub-Saharan Africa.

## The COVID-19 Pandemic and Sub-Saharan Africa: The Need for Point-of-Care Diagnostics

On January 30 2020, the World Health Organization (WHO) declared the coronavirus disease COVID-19 (caused by the Severe Acute Respiratory Syndrome Coronavirus 2 (SARS-CoV-2), in this paper referred to as COVID-19) outbreak as a Public Health Emergency of International Concern, and shortly thereafter called for research on point-of-care (POC) *in-vitro* diagnostics (IVDs) for use at the community level (1). In response, numerous POC IVDs are in development or have entered the market, many of which are so-called rapid diagnostic tests (RDTs) (2).

WHO recommends nucleic acid amplification tests (NAAT) for identification of COVID-19 infection in triage and the tracing of contacts (3, 4). However, in low-income countries, sub-Saharan Africa (sSA) in particular, molecular testing is frequently only available in central reference laboratories. Moreover, testing capacity is limited, leading to long turnaround times which preclude the use for patients and infection control management (5–8).

RDTs are equipment-free, generate a result in a short time (mostly within 30 min), can be operated at the POC level, and by minimally trained healthcare workers outside central laboratory test facilities (9). As of August 18 2020, the Foundation for Innovative New Diagnostics (FIND) (2) lists 18 SARS-CoV-2 antigen-detection RDTs and 163 SARS-CoV-2 antibody detection RDTs that are currently marketed or in development, of which, respectively, 17 and 155 have regulatory approval by the European Community [Conformité Européenne (CE) mark] and five antibody detection RDTs have approval from the United States Federal Drug Agency (US FDA, Emergency Use List). Countries in sSA have successfully deployed RDTs for HIV and malaria diagnosis (10), adding to the expectation for the implementation and successful roll-out of RDTs for the detection of the COVID-19 infection. However, published evidence of performance of these RDTs so far is limited (see below). Most studies focused on diagnostic accuracy and were carried out in reference settings in high- and middle-income countries early affected by the COVID-19 pandemic (11). By contrast, few studies have assessed POC use and RDT user-friendliness and, to the best of our knowledge, so far none have assessed their integration in the healthcare setting in sSA.

## The Scope of This Paper, Terms Used

The present article aims to pinpoint product- and healthcare-related requirements for the implementation of RDTs in detecting the SARS-CoV-2 infection in the context of sSA. The term “low-resource settings” (LRS) refers to low-income countries (of which 29 out of 33 are located in sSA) (12) as well as to remote and under-served areas in middle-income countries.

The ASSURED criteria [affordable, sensitive, specific, user-friendly, rapid and robust, equipment-free, and deliverable to those who need it (13)] were used to interpret the WHO request for “POC diagnostics for use in the community” (14) and to define the COVID-19 RDT products. Rather than aggregating and comparing diagnostic accuracy of the COVID-19 RDTs, the present review reviews their design (format, package, and configuration), specimen and sampling, usability, robustness, and stability, all in view of the end-user and large-scale implementation in sSA. Where relevant, comparisons are made with the deployment of RDTs in sSA targeting malaria, HIV, and other infectious diseases. Among those listed by the WHO, the testing scenarios considered for the COVID-19 response are (i) case management of suspects (detect active infection, triage), (ii) contact tracing (detect asymptomatic and symptomatic acute infection), and (iii) surveillance (detect acute or past exposure or infection). The scenarios of monitoring response/recovery, tool for prognosis, vaccine response, and environmental monitoring are not addressed (15).

From a communication perspective (i.e., avoiding confusion with the SARS virus epidemic from 2002), the WHO decided to name the disease which was caused by the Severe Acute Respiratory Syndrome coronavirus 2 (SARS-CoV-2) not after the virus; instead the WHO proposed the name “COVID-19 disease” (16). The name COVID-19 has been widely adopted by the scientific community as well as by health authorities and the lay press. For convenience and easy reading, the present text, IVDs and RDTs for COVID-19 disease are therefore further referred to as COVID-19 IVDs and COVID-19 RDTs, respectively, with antigen-detection and antibody-detection RDTs written as COVID-19 Ag RDTs and COVID-19 Ab RDTs. When referring to the virus or IVD brand names, the term SARS-CoV-2 is used.

## Search Strategy

We have reflected on COVID-19 RDTs that are currently being developed and marketed. Guidelines and policy briefs from international organizations [WHO, Africa and Europe Centers for Disease Control and Prevention (Africa CDC, ECDC)], US FDA, International Medical Device Regulators Forum) and published literature (English and French language) were searched for the implementation of RDTs in response to COVID-19 and the control of other infectious diseases in sSA (malaria, HIV, cholera, respiratory tract viruses). Selected items were further explored by the snowball strategy using PubMed and gray literature, complemented with our own field observations.

To assess the published evidence about COVID-19 RDTs, a literature search was performed on PubMed using the strings “(COVID-19) AND diagnostic” “(COVID-19) AND antigen,” and “(COVID-19) AND antibody.” Based on successive screening of the title, abstract, and full text, original research articles that reported the evaluation of an IVD for COVID-19 were included. Articles assessing COVID-19 IVDs that met the ASSURED criteria were analyzed in detail, excluding non-commercialized products and those which did not evaluate clinical specimens or did not include controls. For each RDT, the following data were extracted and imported into a Microsoft Excel worksheet (Supplementary Table 1): PMID, title, assay type and target (antigen/antibody), brand, authors, citation, product code, and lot number. Technical specifications and product performance characteristics were extracted. Pre-publication papers were not included. The search was last updated on August 10 2020.

For a subset of one COVID-19 Ag RDT product and of 20 COVID-19 Ab RDTs retrieved by the literature search, the instructions for use (IFU) were obtained from the manufacturer's website or by correspondence with the manufacturer. Complementary information about format, configuration, package, eligible specimens, and stability were retrieved from the IFU and added to the worksheet.

To discuss the utility of COVID-19 Ab RDTs in the different testing scenarios, we used published accuracy data from two recent meta-analysis studies addressing COVID-19 Ab RDTs, as one of the studies provided a comparison between RDTs and laboratory-confined antibody testing by ELISA and chemiluminescence assays (CLIA) (11, 17). Data about the review was primarily presented with the number of RDT products (rather than the number of studies) as the denominator.

## COVID-19 Ag RDTS and COVID-19 Ab RDTS: Products and Studies Retrieved

A total of 47 articles on COVID-19 RDTs were retrieved, 42 (89.4%) of them assessed COVID-19 Ab RDTs comprising a total of 54 RDT products. Another 5 (10.6%) studies assessed COVID-19 Ag RDTs, all assessing the two products, i.e., the COVID-19 Ag Respi-Strip (CORIS BioConcept®, Gembloux, Belgium), further shortly referred to as CORIS COVID-19 Ag Respi-Strip and the BIOCREDIT COVID-19 Ag test (RapiGEN Inc. Gyeonggi-do, Republic of Korea) further shortly referred to a BIOCREDIT COVID-19 Ag test. To compare the latter products, the single other POC IVD for COVID-19 Ag detection retrieved in the literature search was used, i.e., the 2019-Novel Coronavirus (2019-nCoV) Antigen Rapid Test Kit (BIOEASY Biotechnology Co., Shenzhen, China), further referred to as BIOEASY 2019-nCoV Ag Rapid Test Kit. The BIOEASY 2019-nCoV Ag Rapid Test Kit is based on immunofluorescence and needs a reader, so does not fit the ASSUR*E*D criteria (*E* = equipment-free).

In addition to the COVID-19 RDTs, a number of low-complexity cartridge-based NAAT platforms (comprising sample preparation, amplification, and signal visualization in a closed format) were identified during the literature search, as well as simplified (e.g., isothermal) COVID-19 based IVDs which are in development (18–20). Although some are promising for POC testing, they are not equipment-free and are thus not discussed here.

All 56 COVID-19 RDTs were based on the lateral flow immunochromatographic test platform comprising a nitrocellulose strip embedded in a cassette or applied in a tube format and with test results presenting as colored lines read by the naked eye.

As to regulation, according to the FIND SARS-CoV-2 Diagnostic Pipeline (2), the CORIS COVID-19 Ag Respi-Strip and the BIOCREDIT COVID-19 Ag RDTs as well as the BIOEASY 2019-nCoV Ag Rapid Test Kit were CE marked. Of the 54 COVID-19 Ab RDTs, 40 (74.1%) were also listed on the FIND SARS-CoV-2 Diagnostic Pipeline; 34 (85%) of them were CE-marked (63.0% of all COVID-19 Ab RDTs), two products had FDA-Emergency Use Approval.

Both the CORIS COVID-19 Ag Respi-Strip and the BIOEASY 2019-nCoV Ag Rapid Test Kit detected the nucleocapsid protein of the SARS-CoV-2 virus. This choice was based on the 2003 SARS-CoV epidemic, which identified the nucleocapsid protein as the best target for antigen detection, with high sensitivity in an ELISA and RDTs (21–24). The nucleocapsid protein is relatively conserved, immunogenic, and abundantly expressed during infection (22, 25). The antigen detected by the BIOCREDIT COVID-19 Ag test was not indicated in the article evaluating the product, the IFU of this product could not be retrieved.

The product specifications of the COVID-19 Ab detecting RDTs retrieved from the published papers and the IFUs are listed in Tables 1, 2, respectively. Over 90% of products detected both IgG and IgM; three of these products had separate strips for both antigens. Products used either recombinant spike or nucleocapsid protein or both as the detection antigen (Table 1). The spike protein is of interest as it is highly conserved and specific and its receptor-binding domain protein (RBD-S) is expected to be neutralizing (25, 26).

**Table 1:** Selected product specifications and study design for 54 COVID-19 antibody detection rapid diagnostic tests (RDTs) retrieved from 45 peer reviewed original research articles.

**Product Specifications Study Design**	**RDT products**	
	**Nr**	**%**
**ANTIBODIES DETECTED**		
° IgG	1	1.9
° IgM	1	1.9
° IgG & IgM	50	92.6
° Total antibodies	2	3.7
**DETECTING ANTIGEN (BINDS ANTIBODIES)**		
° Spike protein	8	14.8
° Nucleocapsid protein	4	7.4
° Spike protein and Nucleocapsid protein	6	11.1
° Could not be retrieved by investigator	4	7.4
° Not mentioned	32	59.3
**SPECIMEN ASSESSED**		
° Serum or plasma only	29	53.7
° Venous whole blood (with/without other specimens)	19	35.2
° Capillary whole blood (with/without other specimens)	5	9.3
° Not mentioned	1	1.9
° Equivalence of claimed specimen types	6	11.1
° Equivalence of claimed anticoagulants	0	0
**ORIGIN OF SAMPLES FROM INDEX PATIENTS:**		
° Hospitalized patients	23	42.6
° Outpatients	7	13.0
° Not specified if in- or out-patients	26	48.1
° Disease severity mentioned	7	13.0
**ORIGIN OF SAMPLES FORM CONTROL PATIENTS**		
° Hospitalized patients	18	33.3
° Outpatients	9	16.7
° Not specified if in- or out-patients	26	48.1
° Disease severity mentioned	1	1.9
**GEOGRAPHIC ORIGIN OF PATIENTS ASSESSED**		
° Asia	12	22.2
° North America	8	14.8
° South America	4	7.4
° Europe	42	77.8
° Australia	5	9.3

*All RDTs are lateral-flow immunochromatography assays. Numbers refer to the number of COVID-19 RDT products*.

**Table 2:** Selected specifications and test characteristics of a subset of 20 COVID-19 antibody detection rapid diagnostic tests (RDTs) retrieved from 20 products' instructions for users (IFU).

**Product specifications**	**Nr**	**%**
**RECOMBINANT DETECTION ANTIGEN**		
° Spike protein	3	15.0%
° Nucleocapsid protein	0	0.0%
° Spike and Nucleocapsid protein	3	15.0%
° Not mentioned	14	70.0%
**FORMAT, CONFIGURATION, PACKAGE**		
° Strip-in-cassette	20	100.0%
° Strip-in-tube	0	0.0%
° Sampling material in kit	4	20.0%
° Transfer device in kit	15	75.0%
° Self-contained kit (containing both sampling materials and transfer device)	20	100.0%
° Controls included in the kit	1	5.0%
**CLAIMED SPECIMENS**		
° Plasma / Serum	2	10.0%
° Serum/ Plasma/ Whole blood	8	40.0%
° Serum/ Plasma/ Whole blood including capillary finger prick blood	8	40.0%
° Serum/Plasma/Whole blood but not recommended for finger prick blood	2	10.0%
**REPORTING OF SENSITIVITY**		
° Sensitivity expressed in function of time since symptom onset	5	25%
**STORAGE TEMPERATURE**		
° 2/4°C up to 30 °C	20	100.0%
**OPERATING CONDITIONS AND IN-USE STABILITY (STABILITY**		
**AFTER OPENING THE DEVICE POUCH)**		
° Operating conditions mentioned	0	0%
° In-use stability 30 min	1	5.0%
° In-use stability 1 h	3	15.0%
° No in-use stability mentioned, “process immediately”	16	80.0%
**SHELF-LIFE**		
° 2 months	1	5.0%
° 6 months	1	5.0%
° 12 months	3	15.0%
° 18 months	2	10.0%
° Not mentioned in IFU	13	65.0%
**RESULT STABILITY**		
° 15 min	5	25.0%
° 20 min	11	55.0%
° 30 min	1	5.0%
° Not mentioned in IFU	3	15.0%
° **SAMPLE STABILITY**		
° Capillary blood finger prick: “perform immediately” (all 8 products)		
° Venous whole blood: 2–7 days at 4–8°C (median 3)		
° Serum/Plasma: 2–7 days at 4–8°C (median 3)		
° Not mentioned for 9 (45%) products		

*All RDTs are lateral-flow immunochromatography assays. Numbers refer to the number of COVID-19 RDT products*.

For two-thirds (36/54, 66.0%) of the products, the identity of the recombinant detection antigen was not mentioned in the article and neither was it mentioned in 70% of product IFUs (Table 2). This proportion is in line with the observation of Pallett et al. (27). They reported that the majority of the 284 COVID-19 Ab immunodiagnostics assessed (of which many had regulatory approval) either made a non-specific reference to the SARS-CoV-2 antigen and antibody targeted (59.2%) or listed no information whatsoever (17.3%) about the nature of the antigen or antibody targeted—these proportions were higher compared to the ELISA platform (27). For the International Medical Device Regulators Forum (28), proprietary information does not need to be disclosed in the IFU. Information about the nature of the antigen and antibody targeted, however, cannot be labeled as proprietary information and is essential for the comparison and monitoring of the diagnostic accuracy but also for the interpretation of seroprevalence studies and presumed immunities (27). In addition, the WHO recommends that the IFU of RDTs should contain enough and detailed information about the test principles including identification of the antibody and antigen and the chemical principles of detection (29).

The origin of patient and control samples were not specified for nearly half of the COVID-19 Ab RDTs assessed and disease severity was only reported for a minority of product evaluations [seven products assessed in four studies (Table 1)]. The origin of patients (hospitalized vs. non-hospitalized) was not reported for nearly half of the products, and details of disease severity were provided for only a few products. Of note, viral load is expected to be higher in hospitalized vs. non-hospitalized patients (30), and sensitivity and test line intensities are lower in mild COVID-19 disease (31, 32). Providing relevant patient information is part of the Standards for the Reporting of Diagnostic Accuracy Studies (STARD) checklists (33) and essential to understand a product's performance in different settings (11). Further, <10% (5/56) of COVID-19 RDTs assessed in our literature review were evaluated in the POC setting and no study was conducted in sSA (Table 1). Among 17 studies evaluating COVID-19 Ab RDTs reviewed in a recent meta-analysis, only two were conducted at the point of care, representing only 2% of the total tests assessed (11).

## Implementing COVID-19 RDTS for Sub-Saharan Africa: The RDT Product

### Specimen and Sampling

#### COVID-19 Ag RDTs

So far nasopharyngeal secretions are the preferred specimen for COVID-19 Ag RDTs as well as for NAAT reference testing (34). Specimen equivalence of the CORIS COVID-19 Ag Respi-Strip has been evaluated in one study, demonstrating equivalence of nasopharyngeal aspirates, and nasopharyngeal swabs (21). In addition, the product's IFU mentions nasopharyngeal washes as an eligible specimen—however, this information was not supported by published evidence. The BIOCREDIT COVID-19 Ag test was evaluated on saliva, nasopharyngeal swabs, nasopharyngeal aspirates, throat swab, throat swabs, and sputum (35). The BIOEASY 2019-nCoV Antigen Rapid Test Kit has published an evaluation of nasal/nasopharyngeal swabs and oropharyngeal swabs as eligible specimens (36); the product's IFU in addition mentions sputum as a specimen with no published data referred. Given patients' reluctance for diagnostic sampling in LRS (37), alternative specimens (such as saliva) would be more acceptable than a nasopharyngeal swab or aspirate (38, 39). COVID-19 has been detected in self-collected saliva samples using NAAT methods but this needs further study (40, 41).

For COVID-19 antigen detection, sample stability is a concern: in the studies published on the CORIS COVID-19 Ag Respi-Strip, the BIOEASY 2019-nCoV Antigen Rapid Test Kit, and the BIOCREDIT COVID-19 Ag test, samples were kept at 4°C or −70°C when testing could not be done immediately, which indicates the need for a cold chain (21, 35, 36). The IFU of the CORIS COVID-19 Ag Respi-Strip indeed confirms the need for freezing at −20°C if immediate testing of the sample is not possible and mentions a loss of signal intensity when samples are stored at 4°C. By consequence, sample stability of the COVID-19 Ag RDTs is a concern. As a comparison, the WHO draft specifications for COVID-19 POC IVDs deployable at triage list as a minimum (“acceptable”) requirement a pre-testing sample stability of 30 min at 10–35°C, 2–4 h at 2 to 8°C and 8 h in a generic preservative at 2–8°C (39).

To facilitate logistics and prevent patients being lost to follow-up, the sample or sample-buffer mixture for the COVID-19 antigen testing should be appropriate for downstream NAAT-testing (sufficient volume, RDT buffer compatible with the NAAT assay, preserved stability, and contained in a leak-free tube). In the publications on both the aforementioned COVID-19 Ag RDTs the same sample was used for NAAT and Ag detection, indicating the possibility of downstream NAAT (21, 35, 36).

#### COVID-19 Ab RDTs

For COVID-19 Ab RDTs, finger prick capillary blood specimens stand out as the preferred specimen (6, 11, 42), as finger pricks are minimally invasive and safe and easy to perform. In addition, in sSA, healthcare workers and patients are familiar with finger prick sampling, particularly in malaria-endemic areas. Probably explained by the use of stored (left-over) samples, published evaluations of the COVID-19 Ab RDTs were done on only serum or plasma for half (29/54 products, 53.7%) of the COVID-19 Ab products; 19 (35.2%) were also evaluated on venous blood and five (9.3%) on capillary whole blood, all of them in a POC setting (Table 2). Only eight COVID-19 Ab RDTs (14.8%) in four studies have published evidence about equivalence of venous whole blood with serum or plasma (31, 42–45). In these studies, plasma was obtained by centrifugation of EDTA whole blood and over 97% agreement was found between both specimen types. Only one article (assessing a single product) studied specimen equivalence between plasma, venous whole blood, and finger prick blood and found no difference in the 10 paired samples (seven COVID-19 patients and three healthy controls) assessed (29). Although so far venous whole blood and serum appears to be equivalent, further study is needed to validate the specimen equivalence, as serum and plasma are expected to have higher antibody titers compared to whole blood (11). None of the studies retrieved had assessed the equivalence of different anticoagulants (Table 2).

Specimen type may affect diagnostic performance of RDTs: as an example, for HIV 1/2 RDTs, higher numbers of false positives in whole blood as compared to plasma specimens were shown (46). Further, the concentration of antibodies is higher in serum and plasma than in whole blood, which may lead to differences in sensitivity and specificity if the same volume is used (47). In their IFUs, all 20 COVID-19 Ab RDTs mentioned both serum and plasma. Two products had only plasma and serum mentioned as eligible specimens in their IFU, and two products indicated the use of serum, plasma, and whole blood but specified that finger prick blood was not recommended.

### ASSURED: Sensitivity and Specificity of the COVID-19 RDTs, Utility in Testing Scenarios

#### COVID-19 Ag RDTs

At reference testing, the specificity of the COVID-19 Ag RDTs was 100% in all studies for both products (21, 36, 48–50) but diagnostic sensitivity was low for the CORIS COVID-19 Ag Respi-Strip: sensitivity was 82–100% for samples with high viral load but overall sensitivity ranged from 24 to 58% (21, 48–50). The BIOEASY 2019-nCoV Antigen Rapid Test Kit showed a higher overall sensitivity (95%), which however declined to 72% in patients with low viral loads (36). The higher sensitivity may be explained by the fact that the fluorescent signal was detected by equipment as compared to a colorimetric reading by the naked eye in the case of the CORIS COVID-19 Respi-Strip.

The pattern of moderate sensitivity/high specificity of the CORIS COVID-19 Ag Respi-Strip is comparable with those of influenza RDTs (51) and its consequences are twofold. Firstly—provided confirmation of the high specificity in large prospective series—a positive test result can be confidently accepted as a diagnosis of acute COVID-19 infection. Secondly, given the low sensitivity, negative test results imply referral of the patient (or sample) for subsequent NAAT testing (21). In a similar scenario in Kenya, influenza Ag RDTs have been proposed for surveillance and even clinical management in remote settings where capacity is limited (52). Although COVID-19 Ag RDTs would be of benefit in a triage scenario [short time-to-result, cost-saving, alleviating central testing (21)], the sensitivity of the CORIS COVID-19 Ag Respi-Strip is below the required sensitivity for a decentralized stand-alone POC triage (≥70% acceptable, ≥80% desirable) (39). Furthermore, an even higher sensitivity (≥95% acceptable, ≥98% desirable) is needed in the scenario of COVID-19 contact tracing and diagnosis of cases with subacute infection, as both viral load and pre-test probability (prevalence) are lower compared to the triage setting of acute symptomatic patients (15).

By consequence, sensitivity needs to be improved while maintaining a high specificity, as has been achieved for influenza Ag RDTs and potentially the BIOEASY 2019-nCoV Antigen Rapid Test Kit (53) by optimization of test chemistry and signal detection through digital reading equipment.

In order to use COVID-19 Ag RDTs as a tool to demonstrate viral clearance after recovery (e.g., for reasons of infection control or safely resuming work), further data about the SARS-CoV-2 antigen and viable virus dynamics during the COVID-19 infection are needed. The WHO interim guidelines for COVID-19 laboratory testing (March 2020) (54) do not mention COVID-19 Ag tests for any testing scenario. In a scientific brief, the WHO did not recommend COVID-19 Ag RDTs for patient care (55). FIND mentions the possibility of using COVID-19 Ag tests for case management in high prevalence and active outbreak settings, i.e., (i) at triage (with confirmatory molecular testing of negative samples), (ii) to monitor active infections as well as (iii) in contact tracing (56).

#### COVID-19 Ab RDTs

Data about diagnostic performance of COVID-19 Ab RDTs were recently aggregated in two independent meta-analyses, both including data from peer-reviewed as well as pre-printed articles (11, 17). In addition, a Cochrane review concluded at the end of April 2020 assessed COVID-19 antibody detecting immunoassays but without stratifying for COVID-19 RDTs (30). An overview of COVID-19 antibody kinetics can be found in references (13, 26, 57–59). Briefly, IgM antibodies appear 5–10 days after the first day of symptoms, closely followed but sometimes overlapped by IgG antibodies. IgG and IgM antibodies increase during week 2 and peak in week 3, mean times for seroconversion (60) are 9–11 days after symptom onset for total antibody, 10–12 days for IgM and 12–14 days for IgG. Levels of IgM decline from week 5 onwards and are almost non-detectable by week 7 (26).

For the detection of IgG and/or IgM, both RDT meta-analysis studies computed for COVID-19 Ab RDTs in similar pooled sensitivities of 64.8 and 66.0%, which were much lower than the corresponding sensitivities of 97.8 and 84.3% for the laboratory-confined CLIAs and ELISAs, respectively (11). Pooled sensitivities of IgG (65%) and IgM (62%) were almost identical—precluding their differential use in diagnostic algorithms—and increased in parallel during the course of infection: in week 1 post symptom onset, aggregated sensitivity for IgM and IgG was 25.3 and 13.4% respectively, increasing to 51.8 and 50.1% in week 2 and exceeding 70% only from week 3 onwards (69.9 and 79.8%, respectively), with high variations between different products (11). Pooled specificity for COVID-19 Ab in the study of Bastos et al. was high (96.6%) but lower compared to ELISAs (99.7%) (11); Ricco et al. computed a specificity of 98.0%, respectively (17). However, as mentioned by the authors, specificities might be biased by the case-control design used in most studies (11, 17) as well as by reporting bias—i.e., exclusion from publication of products with low specificity (17).

The sensitivity and specificity findings of the studies retrieved in our literature review (assessed for 28 articles published until June 2020), are in line with the above: combined IgG/IgM sensitivity ranged from 42.3 to 100.0% and specificity from 89.2 to 100.0%. In addition, in these articles, we looked in detail at the control panels used for assessing specificity: they included other coronaviruses (SARS-CoV, NL63, HKU1, 229E, OC43), cytomegalovirus, Epstein-Barr virus, severe fever with thrombocytopenia syndrome virus, dengue virus, human hepatitis B virus, *Mycoplasma pneumoniae*, parvovirus infection, *Bartonella henselae, Brucella spp*., and autoimmune pathologies. Apart from dengue, no other tropical disease was evaluated for potential cross-reactions and only few products were challenged with HIV 1/2 positive samples. Of note, tropical diseases such as malaria, dengue, schistosomiasis, and sleeping sickness have been associated with false positives in antibody detection RDTs for HIV1(/2) and malaria (61–65) but have, to the best of our knowledge, not yet been assessed for cross-reactions with COVID-19 Ab RDTs. Assessing COVID-19 Ab RDTs for contextual pathogens in sSA is urgently required (6) and the WHO requirements for Emergency Use Listing (see below) of COVID-19 Ab RDTs lists HIV and malaria among the list of organisms to be tested for cross-reactions (66).

Recommendations about the use of COVID-19 Ab IVDs in general (i.e., all diagnostic platform comments) are as follows: WHO interim guidelines for COVID-19 clinical management state that COVID-19 Ab IVDs have no place in the diagnosis of current COVID-19 infection (triage scenario) (4) except for patients presenting late who may have negative NAAT results. In these cases and provided there is a strong epidemiological link to COVID-19 infection, paired serum samples (in the acute and convalescent phase) can support diagnosis through the demonstration of seroconversion (5, 67). Further, COVID-19 Ab IVDs can be used in the case of sero-epidemiological studies (which define levels and geographic extent of population exposure) (68). COVID-19 Ab tests should not be used as criteria to discharge patients from hospitals (as the presence of antibodies does not mean “non-infectivity”) nor as criteria for (health care) workers to return to work [as the presence of antibodies does not mean “protection” (6, 69)]. Further, population screening in low prevalence settings is not recommended, as it will probably result in more false-positive than true positive results (60)—this will be particularly the case of RDTs given their lower specificity.

As for COVID-19 Ab RDTs, in a scientific brief from April 2020, the WHO recommended them only for research settings but not for patient care (55). Given the poor performance (in particular sensitivity of COVID-Ab RDTs compared to ELISA and CLIA platforms, both aforementioned meta-analysis studies share this conclusion and question the utility of using (or continuing to use) COVID-19 Ab RDTs for medical decision making (11, 17). For seroprevalence studies, the WHO mentions the option of COVID-19 RDTs, provided confirmatory testing by ELISA and with serum as the preferred specimen (68). FIND mentions the use of COVID-19 Ab RDTs for screening of contacts ≥10 days post exposure (56). The interim guidance on COVID-19 Ab RDTs from the African Union, Africa CDC, and WHO Africa (June 2020) also mentions three indications, particularly for areas with limited or no access to NAAT (6): COVID-19 Ab RDTs can be used as an initial screening at POC triage (with sampling patients who tested negative for molecular testing), screening for contacts (also with molecular testing of negative contacts), and surveillance (sero-epidemiological studies). In a viewpoint paper, the authors refer to the latter scenarios for the successful deployment of COVID-19 Ab RDTs in triage (Peru) and contact tracing (Singapore) (60).

### Test Format, Configuration, and Package

#### COVID-19 Ag RDTs

**Figure 1:**
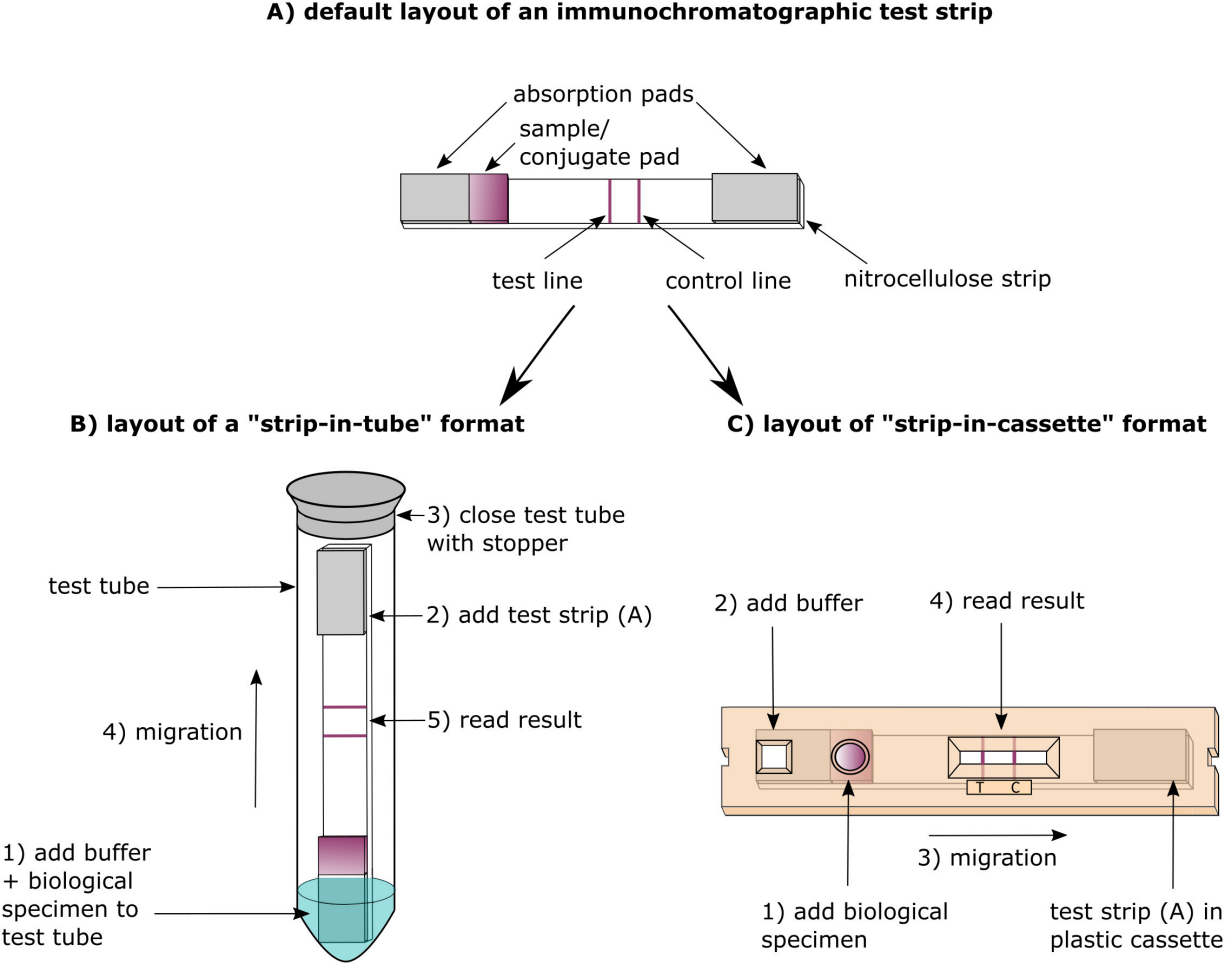
Design and principle of antibody vs. antigen detection lateral-flow immunochromatography assays (LFIA).

The strip-in-tube format of the CORIS COVID-19 Ag Respi-Strip is less suitable to POC testing compared to the strip-in-cassette format which is preferred by healthcare workers performing malaria diagnosis (70) (Figure 1). Compared to the cassette, the tube format is more difficult to manipulate and writing the patient's identification is challenging (not enough space on the strip, a felt pen is needed to write on the tube). Moreover, there are biosafety issues: in similar strip formats, we demonstrated viable bacteria on processed cholera RDT strips (71). The BIOEASY 2019-nCoV Antigen Rapid Test Kit uses a cassette format, but sample preparation is at a similar level of complexity as the “strip-in-tube” format: the swab has to be inserted and mixed into a dropper bottle and next the mixture is applied from the dropper bottle to the cassette.

Sampling material is not included in either of the COVID-19 Ag RDTs we analyzed. However, despite adding to the cost, a “single pack” format (containing everything for a single sample test) could be more convenient for decentralized testing and in addition ensure the use of the correct buffer and buffer volume (72). Humidity in tropical countries accelerates RDT deterioration (70) and therefore a humidity-indicating desiccant should be added to the RDT strip package (73). Given their impact on sampling, the recommended flocked swabs for COVID-19 Ag RDTs (providing a higher volume uptake than conventional swabs) with an aluminum or plastic shaft (74, 75) should be included in the package and categorized as a kit component (i.e., essential to the RDT) rather than as an accessory (i.e., a replaceable item) (76).

#### COVID-19 Ab RDTs

All COVID-19 Ab RDTs we analyzed were based on the strip-in-cassette format (Table 2). While Africa CDC states as one of the advantages of COVID-19 Ab RDTs that they often include all the materials needed to perform the test including sampling materials and sample transfer devices (capillary tube or pipette) (6), finger prick material (lancets and alcohol swabs) were included in only 4/20 COVID-19 Ab RDT products for which the IFU was assessed. In the case of the FaStep COVID-19 IgG/IgM Rapid Test Device (Assure Tech. Hangzhou Co., Ltd, Hangzhou, China) the supply of finger prick material was especially confusing as only serum and plasma were listed as eligible specimens in its IFU. Five COVID-19 Ab RDTs did not include a sample transfer device in the test kit, requiring a micropipette to be present on site. The use of the sample transfer device provided in the kit, which is calibrated for a certain volume, can be problematic when transfer volumes for plasma/serum and whole blood are different, as was the case for Zheijang Orient Gene Biotech (Huzhou, China), where 5 μl of plasma/serum but 10 μl of whole blood should be applied and only a single transfer device was included.

#### Controls—Waste Management

In lateral immunochromatographic RDTs (such as COVID-19 RDTs), the integrated control line only confirms migration of the sample-buffer-conjugate along the nitrocellulose strip and does not include a check for the antibody-antigen interactions. For some (2/22, 9.1%) of the COVID-19 RDT products, lyophilized negative and positive controls were available but should be procured separately. In LRS, the inclusion of positive and negative controls within the RDT test kit itself is however an asset and is also listed as “desirable” in the WHO draft specifications for COVID-19 POC IVDs (39). Among the IFUs assessed, only a single product provided positive controls in the test kit: the StrongStep SARS-CoV-2 IgG/IgM Antibody Rapid Test (Liming Bio-Products, Jiangsu, China).

Finally, as is the case for other RDTs, materials of components, package, and accessories should be compatible with local waste management capacities such as field incinerators; compostable plastics are an asset for minimal environmental impact (39, 77).

### ASSURED: Usability, Robustness, and Environmental Stability

#### Usability and Robustness

In addition to complying with STARD guidelines (33), studies should actively observe and assess the product's usability. Usability studies—also referred to as ease of use or user-friendliness studies assess the product design and IFU as to be understood and manipulated by the intended user. Usability studies are an essential part of IVD development (78); originally most encouraged for RDTs used for HIV-self testing, the WHO also recommends them for other RDTs such as syphilis, hepatitis B, and hepatitis C (9).

For usability studies of RDTs in LRS, the WHO recommends the inclusion of label comprehension studies, result interpretation studies, and trained user observations (9). Usability studies should address the intended user (representative for level of education, literacy, auxiliary skills, and language) in the usual setting and with the RDT product as marketed, i.e., with the components, accessories, and IFU as supplied with the RDT product (29). Depending on the target or disease program, intended users of RDTs in LRS may be clinical healthcare workers and trained lay providers (such as in the case of malaria and cholera) (29). Alternatively, some RDTs—although conceived for POC testing outside the laboratory—are mainly used within (basic) laboratories and with laboratory technicians as the user—an example RDTs used for influenza diagnosis (79). Given the surveillance component of the COVID-19 response, this may be the case for part of the COVID-19 RDTs, too.

Robustness (sturdiness) of the RDT is measured in so-called flex studies which study the RDT performance while mimicking procedural (user) errors (such as adding too few or too much sample volume) and harsh environmental conditions for storage (humidity, light, temperature). Usability and flex studies identify and mitigate potential user-related hazards, orient training and supervision needs, improve workflow and ergonomics, and promote integration of the IVD in the healthcare system (79).

Table 3 lists topics of product- and user-related factors that may influence user-friendliness of RDTs and may be assessed in robustness studies. Both usability and flex studies cross-reference with product specifications and analytical performance studies: as an example, inter-operator agreement of test and control line readings (precision testing) is related to product characteristics such as the presence of crisp and clear test lines. Labeling and IFU including accessible “bench-aids” or quick reference guides and should anticipate user- and product-related failures that cannot be mitigated by design. To be effective, IFUs should be adapted to the literacy and performance level of the user working in stressful conditions (84, 85).

**Table 3:** Rapid diagnostic tests applied in low-resource settings: examples of factors related to ease of use (user friendliness, usability) or robustness.

**Product specifications/characteristics**	**Human (user) factors—comments**
**FORMAT, PACKAGE, AND CONFIGURATION**	
Format: ° Strip-in-cassette vs. strip-in-tube Configuration: ° Kit with individual tests (*n* = 25), transfer devices, and 1 buffer vial ° Self-contained kit which also contains sampling materials • Alcohol swabs, finger prick lancets (Ab tests) • Flocked swabs, transport medium (Ag tests) ° Positive and negative controls available in the test kit Package: ° Single pack: individual tests packed with small dedicated buffer vial	° Cassette more familiar and preferred to tube (similarity with malaria and HIV RDTs) but in the case of COVID-19 Ag RDTs, the use of cassette may increase the number of steps including sample transfer ° Self-contained kit easier for field testing (procedure, logistics) ° Single pack assures correct use of buffer vial and volume
**SPECIMEN TYPE, SAMPLE COLLECTION, AND SAMPLE STABILITY**	
° Biological specimens (validation of specimen types/anticoagulants) • COVID-19 Ag RDTs: upper respiratory tract specimen - nasopharyngeal swab, oropharyngeal swab, saliva • COVID-19 Ab RDTs: - serum, plasma, whole blood: venous vs. capillary ° Biosafety aspects of sample collection ° Sample stability • COVID-19 Ag RDTs: samples need to be frozen if immediate testing is not possible ° Shelf-life	° COVID-19 Ag RDTs: • Upper respiratory tract sampling is challenging for laboratory technicians [influenza, (79)] • Suboptimal sampling is associated with false-negative results (80) • Self-collected saliva is associated with false-negative results (81) ° COVID-19 Ab RDTs: • Capillary blood is the most common specimen for malaria and HIV testing • Most RDTs have been validated on serum/plasma only • Test kit must be adapted to match serum/plasma vs. whole blood (e.g., samples transfer device)
**DEVICE COMPONENTS AND ACCESSORIES**	
° Device (cassette, tube) easily writable, large read-window ° Transfer device: easy to handle, self-regulating, stable volume mark ° Lancets: auto-retractable, painless ° Alcohol swabs: large, with enough content ° Desiccant with humidity indicator	
**OPERATING CONDITIONS**	
° Environmental temperature ° Relative humidity ° Light	° Particularly relative humidity is harmful as it affects the nitrocellulose strip and the applied antigens/antibodies ° Poor light conditions (evening and night shifts) hamper visual detection of faint test lines, particularly in staff with presbyopia
**PROCEDURE**	
° Time to let the RDT adjust to room temperature ° Numbers of steps (particularly timed steps) • Collecting/Preparing specimen: ≤ 1 operator step preferable • Assay performance RDT: ≤ 2 timed steps preferable ° Transfer of sample volume: • not too small (tendency to apply too much) • nor too high (sampling, transfer) • easy use of transfer device (large enough, visible volume mark) ° Process time (time-to-result) ° Hands-on time	° RDTs with storage temperature <30°C are frequently stored in the refrigerator ° Error-prone procedures (in particular when self-testing) include • Transferring sample to cassette or tube • Adding volumes of sample and/or buffer: tendency to add too high volumes • Mixing buffers vials from different production lots (or products) ° Too-long process time incites too early reading
**STABILITY**	
° Storage temperature ° Open pouch stability, in-use stability (stability once the package has been opened)	° Stability up to 30°C only implies the need for a “cool chain” In-use stability is important (relative humidity)
**READING AND INTERPRETATION**	
° Swift migration with excellent background clearance of the strip ° Crisp, high intensity test lines (no faint or blurred lines) ° No ghost lines (application site of control/test antibody lines) ° Low frequency of invalid results (i.e., absence of control line or control line obscured by blood-buffer mixture) ° Clear instructions for interpretation ° Extended stable read time (e.g., 60 min duration of valid result) ° Low proportion of invalid test lines ° Low proportion of faint/weak line intensities	° User errors: • disregarding faint test lines as negative and ghost lines as positive • not recognizing invalid tests and anomalies • reading too early (false-negatives) or too late (backflow, false-positives) • interpret test line intensity as indicative for antigen concentration (and clinical severity) ° Product errors: anomalies (70) • poor background clearance blurring test/control lines • incomplete migration • high numbers of absent control lines
**LABELING AND INSTRUCTIONS FOR USE**	
° Visibility (lay-out and presentation) ° Readability (grade of education needed for comprehension) ° Clear and easy-to-understand instructions and labels ° Real-life pictorial instructions is an asset (29)	° Instructions for use must anticipate users' errors ° Translations and understanding of international symbols need to be validated. (82) Complementary support documents: bench aids, flyers, videos…

*According to references (9, 29, 39, 61, 73, 77, 79–81, 83)*.

The CORIS COVID-19 Respi-Strip has been assessed for user-friendliness with a European context method [Scandinavian Evaluation of Laboratory Equipment for Point of Care testing (SKUP, https://skup.org/)], based on satisfactory interviews and ratings (21) expressed by laboratory technicians (21). In addition, proportions of weak test line intensities were recorded (33.0 and 12.3% in two studies, respectively) (21, 49) as well as the proportion of invalid test results (1.5%) and inter-observer agreement of result readings (98.3%) (21).

Table 4 lists findings of usability retrieved for the 54 COVID-19 Ab RDTs retrieved in the literature review. Of note, only a few of these studies (e.g., a study evaluating RDT products for self-testing) (92) were designed specifically for usability testing, whereas other studies reported product-related ease-of-use anecdotally observed alongside diagnostic accuracy evaluations. Despite the scarce and fragmented data and despite the fact that none of these studies addressed the LRS user, some observations are relevant for implementation. Firstly, usability differed between the selected products with most performing well (by trained laboratory staff) but some performing poorly, showing high proportions of invalid test lines (>40%) or invalid and inconclusive test results (>20%). Secondly, migration of the blood-buffer mixture was a substantial problem in certain products and affected test line reading which lead to a high proportion of invalid results. Thirdly, sampling and sampling transfer were confirmed as difficult procedure steps. In addition, incidental shortcomings were observed for instance in the buffer vial (spills, buffer) and IFU.

**Table 4:** Usability (ease-of-use, user-friendliness observations as assessed for 54 COVID-19 antibody detection rapid diagnostic tests (RDTs) retrieved from 45 peer reviewed original research articles.

**Product specifications study design**	**Nr (%) of products assessed**	**Main findings—comments**
° Line intensities	24 (44.4%)	° Presence of weakly colored test lines was reported in nine articles for 24 products • No further details about line intensity provided in three papers (86–88) • Proportions of very weak or weak test lines as 40.1 and 76.9% (27, 31, 89) • Correlation of line intensity with the time since onset of symptoms (two products) (90) • No difference in the number of weak test lines in whole blood vs. plasma (45) • Whole blood compared to plasma/serum: IgM band fainter, IgG line slightly stronger intensity (31) • More faint test lines observed for IgM compared to IgG (31) • Lower line intensities in patients with mild disease compared to severe disease (31) • More weak test line results in critical than mild-moderate cases (89)
° Inter-operator agreement result reading	8 (14.8%)	° Inter-operator agreement was evaluated in three articles for 8 products. • Agreement of 100% among laboratory scientists, four products (45) • Agreement between trained evaluators 95.3% (91) • Agreement between lay volunteers (home-testing) and health professionals (92) • - Product 1: 62.8% for positive tests, 100.0% for negative, and 98.5% for invalid tests • - Product 2: 93.9% for positive tests, 97.0% for negative, and 98.4% for invalid tests
°Anomalies assessed	1 (1.9%)	° A pink background was reported in 1 paper for 3/11 products assessed (32)
°Ease-of-use of components and accessories	13 (24.1%)	° Ease-of-use components and accessories was assessed in 2 studies for 13 products (32, 92). ° Tollanes assessed 11 products in a reference setting (Norway) (32) • User: biomedical laboratory scientists, Method: no data provided • 2/11 products were less user-friendly at test performance and result reading/interpretation, • both products had also higher proportions of invalid/inconclusive results (16.0% and 23.0%) • 2/11 products were less user-friendly at result reading/interpretation • One of them had a high proportion of invalid/inconclusive results (21.0%) • Observations: • -Colored/strong pink background obscuring weak line intensities (three products) • -Blood drawn up to the IgM test lines (two products) • -Buffer vial spills easily, need for pre-analysis mixing of blood and buffer (two products) • -Air bubbles in buffer vial (1 product) ° Atchison 2020 (92) (UK) assessed usability and acceptability of two products for home-testing (self-testing) • Significant usability issues with lancet and transfer pipet (transfer of blood into the sample well) • (Insufficient volume applied) • Minor problems with buffer vial (design) • Problems in migration across the reading window • Instructions for use (interpretation of results) not clear • Blurred photographs made by the participant • Not completing the test (2.5%): putting blood/buffer in the wrong well, spilling buffer, damaging the test)
°Proportion of invalid test results	20 (37.0%)	° Proportions of invalid test results were assessed in 6 studies for 20 products ° For four products—all assessed on plasma and serum, proportions of invalid tests were 0–0.1% (27, 86, 90, 91). ° For 11 products assessed on EDTA-anticoagulated venous blood (32), invalid or inconclusive results were absent or very low (<1%) for eight products, but 16/0, 21.0, and 23.0% the remaining three products, mostly caused by insufficient background clearing (see above) ° For two products assessed for self-testing [see above (92)], invalid results were of 4.8 and 7.4%
° Other investigations	1 (1.9%)	° One study demonstrated a prozone effect for 1 product (93)
**STABILITY TESTING:**		
° In-use stability ° Sample stability ° Result stability	0 0 2 (3.7%)	° Result stability was assessed in one study assessing two products (27) • Result was visible and stable up to 2 h after processing • At 24 h post-test reading, changes were noted in both products (8.8 and 9.8% of tests done), mostly from negative to positive, some tests became unreadable, and few changed from positive to negative. ELISA results were concordant with initial readings at 15 min
**FLEX/ROBUSTNESS TESTING**		
° Flex/robustness study done ° Label comprehension study ° Results interpretation study ° Observed untrained user study ° Other usability study performed	1 (1.9%) 0 1 (1.9%) 0 2 (3.7%)	° A flex study was conducted for one product (31) • Dilutions of samples were used to assess semi-quantification of RDT • Whole blood was intentionally applied without adding buffer (to mimic POC user error) ° An observed untrained user study was performed on two products for home (self-testing) (92) • Online discussions, questionnaires, observations, and interviews of people who tried the test at home • Nationally representative survey of adults in England using the two products at home: the survey showed limitations with the usability of kits. Most people reported completing the test; however, they identified difficulties with practical aspects of the kit, particularly the lancet and pipette, a need for clearer instructions and more guidance on the interpretation of results (see above)

#### Stability

More than in high-resource settings, stability is an issue in tropical LRS. As to storage stability, unlike for instance malaria RDTs [of which many are stable up to 40°C (70)], all but one of the COVID-19 RDTs mentioned 30°C as the maximum storage temperature (the BIOCREDIT COVID-19 Ag test claims stability up to 40°C): this “cool storage” (39) is easily surpassed in tropical climate zones. For COVID-19 POC testing at triage, the WHO draft Target Product Profile lists as an acceptable and desirable target a shelf life of 12 months when stored at 30°C and of 18–24 months when stored at 40°C, respectively, at a relative humidity of 75 ± 5%. Required acceptable and desirable operating conditions (i.e. at the POC when performing the test) are 15–35°C at 25–80% humidity and 10–40°C at 25–90% humidity respectively (39)—none of the IFUs however mentioned operating conditions (Table 2).

Shelf-life was only retrieved from the IFUs for seven COVID-19 Ab RDTs, with three and two products reaching 12 and 18 months, respectively. In a comment, WHO mentions that COVID-19 IVDs “crosscuts cultures, climates, and economies” and acknowledges that the proposed stability and shelf-life requirements do not meet the conditions from tropical countries but encourages manufacturers to develop IVDs resistant to the environmental conditions in tropical countries (39). Further, it should be noted that, in view of the recent accelerated development and production of COVID-IVDs, few stability studies have been conducted. Storage conditions and shelf-life are inferred on extrapolations of small-scale accelerated stability testing design.

In-use stability (i.e., stability of the device (cassette) once the package is opened) was mentioned in the IFUs for only four COVID-19 Ab RDTs and was, respectively, 1 h for three of them and 30 min for the remaining product, much lower than the 1 and 4 h set as acceptable and desirable by the WHO draft Target Product Profile for COVID-19 IVDs (39). A similar observation was made for the result stability (i.e., the stable and readable presence of test and control lines beyond the read time): result stability mentioned for 17/20 COVID-19 Ab IVDs was consistently ≤ 30 min (Table 2), compared to ≥60 min listed as the desired specification for a frontline RDT differentiating bacterial and non-bacterial infections in LRS (77).

### Implementation Monitoring

Unlike laboratory-based immunoassays such as ELISA assays, RDTs have no wash or dilution steps making them vulnerable to non-specific reactions (false-positives) and prozone effects (false-negatives) (94, 95). As noted above, tropical diseases and immunological conditions with low prevalence may cause false positive results (61, 62). Further, incidental product anomalies or malfunctioning may occur. To capture such rare events, some of which may be product related, consistent implementation monitoring is needed. The same goes for user errors and poor practices which can be traced only by regular exchanges inside a laboratory network and through supervision visits and vigilance. Here, the role of national reference laboratories (NRL) and the tiered national laboratory network is pivotal: NRLs should take the lead in selection, distribution, quality control, training, supervision, communication, and post-market surveillance (see below) (96, 97).

## COVID-19 Antigen-Detection RDTS for Sub-Saharan Africa: Integration Into Healthcare

### Target Product Profile—A First Step

The COVID-19 RDTs have been developed for decentralized use in high-income countries. To fit the context of sub-Saharan Africa, a Target Product Profile (TPP) should be defined. TPPs include intended use, target population, diagnostic performance, operational characteristics, throughput, need for batching and turnaround time, as well as training needs, shelf-life, environmental stability, price, and after-sale support (98, 99). The involvement of multiple stakeholders is needed: laboratory staff and frontline healthcare workers, but also manufacturers, health policy makers, and regulators. Examples of TPP for IVDs in LRS have been published recently (77, 100) and the WHO recently published a drafted a TTP document for COVID-19 IVDs RDTs in different testing scenarios (see 5.2) (39). TPPs are living documents and flexibility must be built in to exploit upcoming data about virus dynamics, clinical presentation, and changes in the epidemic which may affect prevalence and pre-test probability (39). A TPP also offers the advantages of product harmonization.

### Technology Landscape, Market Landscape, and Independent Product Evaluations

FIND collates a publicly available tracker list of COVID-19 IVDs and has started an independent product evaluation (2). The WHO-initiated independent evaluation “rounds” of malaria RDTs have shown that publication of head-to-head testing results is a valuable guide to procurement but also stimulates improvements in product performance and compliance (101). In our analysis we found that 24 studies (50.0%) compared multiple COVID-19 Ab products and four (8.3%) were conducted in different test centers. In addition, given the fast-moving research in COVID-19, “technology & market landscape,” review documents are welcome: such documents merge research and market needs and opportunities. Examples are those published by UNITAID for priority diseases in LRS, such as the “fever diagnostics technology landscape” (102).

### Manufacturing Capacity and Quality

Depending on the scale, persistence and potential resurgence of the COVID-19 epidemic, sufficient production volumes of RDTs must be foreseen (19). Leading manufacturers of HIV and malaria RDTs have spare production capacity (103) and manufacturing COVID-19 RDT cassette platforms will only require minor modifications to the existing production lines. In the case of COVID-19 Ag RDTs, the inclusion of sampling components (e.g., flocked swabs) and accessories (personal protective equipment) could be a (temporary) bottleneck. The manufacturer should provide evidence for compliance with a stringent quality management system such as ISO 13485.

Lot-to-lot variation is a well-known challenge for immunoassays and may affect performance (104) and has been well-documented for malaria RDTs (70, 96). The WHO has installed a system of pre-market lot-testing which can detect major product failures (96, 105) but such a system is underpowered to detect small changes between lots (106). Control for minor changes between lots will depend on pro-active implementation monitoring (see above), communication between laboratories and manufacturers, and post-market field effectiveness studies (104, 106). Of note, none of the studies evaluating COVID-19 RDTs retrieved in our search compared different lot numbers.

### Market Intelligence and Interventions

A sustainable market is key for a stable supply. Past experience with malaria RDTs showed that a fast scale-up of production combined with downward pricing negatively impacted manufacturing quality (98). Tenders offering multi-year contracts, fixed volumes, and delivery allow manufacturers to plan productions while investing in quality management and innovation (107). Likewise, experiences in HIV RDTs have shown that the selection of multiple RDT products (all meeting the quality standards) increases competition and leads to a more diverse supply base; in addition it supports product development and innovation (103). In line with an economy of scale, the price of the COVID-19 RDTs will decrease at high-volume production. For malaria RDTs, the price in 2016 was <0.30 USD per test (98) but the price in particular for the COVID-19 Ag RDTs will probably be much higher given their more complicated format, components, and packaging. In addition, the development of COVID-19 Ag RDTs is more expensive compared to COVID-19 Ab RDTs (use of monoclonal antibodies vs. recombinant antigen and simpler sampling materials) (19). Further, indirect costs (transport, training, and quality control) of COVID-19 RDTs will be higher as in-country deployment cannot benefit from the logistics of a national vertical disease-control program as is the case for instance for malaria and HIV.

### Stringent but Timely Regulation

IVD regulation safeguards their safety, quality, and performance but many countries in sSA are low-regulated and may rely on regulatory approvals from highly regulated countries. COVID-19 RDTs are currently CE-certified according to the IVD Directive 1998/79. However, this directive is not stringent, e.g., the CE mark is granted upon manufacturer's self-declaration and only minimum performance data are required (108). The new Directive EU 2017/746 (109) (effective from 2022 onwards) is more stringent than the expiring one, but neither covers the specific environmental and human conditions in sSA. On top of that, regulatory processes take time. The WHO Prequalification therefore established the Emergency Use Listing (EUL) procedure for COVID-19 IVDs, allowing fast-track evaluation of performance, quality, and safety (110). So far, the WHO EUL list of approved SARS-CoV-2 *in vitro* diagnostic products (111) (July 10 2020) only comprises NAAT tests, but 13 COVID-19 Ab RDTs are in the process of application according to the WHO EUL weekly update of ongoing applications dated August 11 (112). The Pan African Harmonization Working Party on Medical Devices and Diagnostics strives to harmonize regulation among the sub-Saharan—this will facilitate market entry of IVDs and avoid duplication of field evaluation studies (58).

### In-Country Deployment of COVID-19 RDTs: Integration Into the Epidemic Response

As is the case for other RDTs, in-country approved policies for COVID-19 RDT use needs to be operationalized by strategic plans and by integration in national laboratory networks coordinated by NRL, in order to ensure quality, training, logistics, and monitoring (113). Training needs for a POC test for triage as projected by the WHO draft TPP are 0.5 days (acceptable) to 2 h (desirable) (39). Further, connectivity with the COVID-19 epidemic response is essential for the timely communication of results but also for stock management and field assistance. Likewise, a swift sample to result flow for reference NAAT testing at the central level is needed. The opportunity of automated reading and transmission of RDT results should be explored: apart from enabling real-time reporting and spatial monitoring, it will ease the workload and reduce transcription errors (113, 114).

### Post-market Surveillance

As described above, implementation monitoring—coordinated by NRL—can detect product or supply shortcomings and result in quality improvement (113). End user awareness, low-threshold monitoring by NRLs, and communication with manufacturers are essential. Major shortcomings should be assessed with the National Regulatory Authorities. The WHO has issued guideline documents about the regulatory framework for IVDs and post-market surveillance directed to WHO prequalified IVDs is currently being updated but the guiding principles can also be applied to COVID-19 IVDs (113).

### Communication: Concern About Commercial Promotion of COVID-19 Antibody Tests

Communication with stakeholders is essential (115). Questions should be tackled early on. In particular, the diagnostic algorithm and limitations of the RDTs should be well-communicated (74) and misconceptions should be clarified. It may be difficult to discuss or explain concepts of test characteristics let alone predictive values or serial (orthogonal) testing: On-line “calculators” [such as provided by US FDA (116) and US CDC (117)] are useful to visualize concepts of test utility such as the relation between prevalence, specificity, and positive predictive value.

Another example of correct communication concerns information about RDTs in commercial promotion. COVID-19 Ab RDTs are intensely promoted but frequently their intended use is mentioned only vaguely and diagnostic sensitivity is presented in relation to NAAT reference testing without mentioning the day of sampling since the onset of symptoms or NAAT testing, as was the case for 75.0% of the IFUs that were presently retrieved (Table 4). Such practices may entail a high risk of wrongful use of RDTs—i.e., use to detect infection rather than exposure—and thereby missing ongoing disease (74). In addition, the ECDC reported several COVID-19 RDT devices with fraudulent documentation and unsubstantiated claims (118). Antibody tests with insufficient clinical performance data have been compiled by the FDA in a “removed” list (119) and the WHO and FDA recently warned against falsified COVID-19 IVDs and reagents (75, 120). It can be expected that in low-regulated countries such practices will become more frequent and NRLs should inform healthcare workers and the community.

## Discussion

### Limitations

The present viewpoint review has inherent limitations—firstly, we only searched the PubMed database and retrieved English-language literatures and did not attempt to assess the gray literature nor the pre-print literature (e.g., medrxiv.org). In this way, relevant information may have been missed. Further, the present literature review was narrative and a systematic review and meta-analysis of performance data were not done. Conversely, the iterative approach allowed us to work fast and provide a “snapshot” of the recent situation (August 10, 2020) in a fast-evolving domain.

As to the panel of COVID-19 RDT products retrieved in the literature, it is clear that the actual discussed products represent a minority of the plethora of products marketed or in the pipeline of development. Moreover, in view of potential selection for evaluation and publication bias (17) they may represent the better end of the products. Likewise, the observations published about usability were made based on small sample sizes and in most cases not explored in detail. Finally, as mentioned by two recent meta-analysis studies, heterogeneity among COVID-19 IVDs is high (11, 17), and aggregated data as listed here probably obscure the better performing products (30).

With regard to implementation in LRS, we did not discuss the role and contribution of certain stakeholders specific to the implementation of IVDs in LRS, such as funders, implementers, technical experts—for a framework analysis and landscape analysis, see reference (121), neither did we discuss funding for diagnostics research and implantation.

### The Way Forward

The multiple shortcomings of the COVID-19 RDTs listed above add to the unsatisfactory low sensitivity of the COVID-19 Ag and Ab RDTs which on its own already refrained experts and policy makers from recommending their use beyond research (4, 11, 17, 55, 67). Considering the WHO COVID-19 Research Roadmap (in which rapid POC IVDs figure at the first priority), leading international experts made a well-motivated plea to use COVID-19 Ab RDTs in areas where NAAT testing is not scalable or affordable, for the scenarios of triage, contact tracing, and surveillance (60). As for COVID-19 Ag tests, the gaps toward utility and implementation in LRS are probably wider, given their low sensitivity (60) but also because of their sample requirements (nasopharyngeal aspirate, biosafety, frozen storage), more complex design, and higher cost. Of note, despite acknowledging these limitations, the CORIS COVID-19 Respi-Strip has been satisfactorily applied in a field setting in the Democratic Republic of Congo as an initial test at triage, with subsequent NAAT testing of patients testing negative (8).

Which would be today's choice for a COVID-19 Ab RDT to be deployed in LRS? The information revealed by the present and prior reviews and publications are too scarce and fragmentary to enable the selection of a “best buy” product. Minimum hints distilled from the above are as follows: look for a product for which the antigen is described and information about specimen equivalence of whole blood is given. Retrieve published evidence about accuracy and have a kit hands-on tested with emphasis on usability (Table 3) and precision, in particular a good inter-reader agreement. A self-contained kit with sampling materials included (provided ergonomic) is an asset. Stick to a single product (to ensure consistency of measurements), build-up good communication with the manufacturer. Monitor lot-to-lot variation and keep records of observations and incidents. If affordable and manageable, validate the RDT product with an ELISA or CLIA as the comparator (68) and build-in repeat (follow-up) sampling at contact tracing and surveillance to anticipate seroconversion delays (60).

Evaluation studies—so far under-powered (11) should be STARD-compliant (11, 33) and address challenges specific to COVID-19 RDTs such as specimen stability (COVID-19 Ag RDTs) and equivalence, product stability, robustness, and usability. Much of the validation work can be done in the reference setting and in head-to-head comparative study designs allowing researchers to preselect best scoring products for field testing. Prospective and well-documented biobanking at the country or regions of implementation is essential (122). Independent analytical and diagnostic performance data of COVID-19 IVDs that are publicly available [such as conducted by US FDA (123) and FIND (124)] must be encouraged (125). FIND invites researchers to submit validation data to a centralized repository accessible throughout the diagnostic community. Of note, practices such as withholding products names in studies—such as in the case of the RDT evaluation by the UK National COVID Testing Scientific Advisory Panel (126) are undesirable. To curb the risk of selective publication [e.g., COVID-19 specificity (11, 17)], the Cochrane systematic review on COVID-19 Ab IVDs makes a plea for the registration of diagnostic accuracy studies in publicly available registers (30). Regulation should find a compromise between the compelling need for POC testing and the scrutiny of product evaluation, documentation, and promotion (25).

## Conclusion

Large scale implementation of COVID-19 RDTs in LRS faces numerous challenges. However, one should not overlook the extremely short period from concept to marketing of the present COVID-19 RDTs (a process which usually takes >5 years) (127) and the high potential for improvements in the short term, as many of the above discussed product shortcomings are easily remediable. Field evaluation studies in LRS should address usability and utility, i.e., integration of best performing COVID-19 RDTs in diagnostic algorithms. As to the integration in healthcare settings, the valuable expertise of the national disease control programs and laboratory networks can be capitalized.

## Author Contributions

JJ and OV had the rationale for this work. JJ did the literature review, writing of the initial draft, and revisions. LH and VK contributed to literature review, revisions, provided critical review, and did the figure design. OL, DA, and OV provided critical review and commentaries. All authors contributed to the article and approved the submitted version.

## Conflict of Interest

The authors declare that the research was conducted in the absence of any commercial or financial relationships that could be construed as a potential conflict of interest.
